# A Review of Current Insights in Fungal Endocarditis

**DOI:** 10.3390/jcm14176149

**Published:** 2025-08-30

**Authors:** Olympia Akritidou, Athanasia-Marina Peristeri, Diamantina Lymperatou, Anastasia Prokopidou, Eirini Christaki, Anna Nikopoulou

**Affiliations:** 1 Department of Internal Medicine, G. Papanikolaou General Hospital of Thessaloniki, 57010 Thessaloniki, Greece; ak.olympia@gmail.com (O.A.); athanasia.peristeri@gmail.com (A.-M.P.); 2 First Department of Internal Medicine and Infectious Diseases Unit, University General Hospital of Ioannina, Faculty of Medicine, University of Ioannina, 45110 Ioannina, Greece; andalimperatou@gmail.com (D.L.); md06996@uoi.gr (A.P.)

**Keywords:** fungi, endocarditis, *Candida*, *Aspergillus*

## Abstract

**Background/Objectives**: Fungal endocarditis (FE), is a rare yet life-threatening disease, which predominantly affects immunocompromised individuals, prosthetic valve recipients, and injection drug users. The purpose of this review is to summarize the evolving epidemiological trends, diagnostic challenges, and treatment strategies, by identifying evidence that supports the optimal clinical approach. **Methods**: A literature search was performed, drawing from sources such as PubMed and Google Scholar and included articles published between January 2015 and March 2025. Clinical studies, case series, and meta-analyses reporting on FE epidemiology, diagnostics, or treatment were included. **Results**: The majority of FE cases is caused by *Candida* species, predominantly *C. albicans*, while *Aspergillus* accounted for a lesser percentage of cases. While blood cultures showed limited sensitivity, adjunctive diagnostic tools such as serum biomarkers (β-D-glucan, galactomannan) and advanced imaging modalities (18F-FDG PET/CT) are increasingly used to guide the diagnostic process. Early surgical intervention combined with antifungals improved survival, particularly for *Aspergillus*, although comprehensive data regarding this approach remains limited due to the rarity of the disease. **Conclusions**: Fungal endocarditis requires an aggressive treatment strategy, integrating early surgery, targeted antifungals, and long-term suppression, especially for prosthetic valves. Despite advances, the complexity of the condition and the variety of the pathogens involved, continue to impede progress towards effective management of FE. Future research must prioritize rapid diagnostics, standardized treatment protocols, and novel antifungals to address this critical condition.

## 1. Introduction

Endocarditis refers to the inflammation of the endocardium, the innermost membrane of the heart, which lines the four chambers and valves. It is a broad term that includes both infective and non-infective causes. Infective endocarditis (IE) refers to the colonization of the endocardium by virulent microorganisms and typically involves the cardiac valves (native or prosthetic), a septal defect, or an indwelling cardiac device [1]. In this review, we will be focusing on infective endocarditis caused by fungal pathogens, which is an infrequent but life-threatening condition.

Fungal endocarditis (FE) is an uncommon yet emerging entity, that constitutes about 2% all IE cases [2]. The prevailing etiological agents are *Candida* spp., that is mostly found in younger populations, followed by *Aspergillus* spp., which is more commonly associated with older age [3,4]. Clinically, FE is presented mainly with constitutional symptoms and subacute onset, thus requiring high index of suspicion for pursuing the diagnosis. Blood cultures are time-consuming and have low sensitivity, making diagnosis of FE even more demanding [2,5]. This diagnostic challenge is depicted in many cases where FE was revealed post-mortem [2,6,7]. Treatment typically involves prolonged systemic antifungal therapy in combination with surgical debridement, highlighting the need for a multidisciplinary team approach in managing patients with this complex condition. Despite the progress that has been made in understanding this clinical entity, the prognosis remains poor with a high mortality rate of around 40% or even higher if endocarditis is caused by *Aspergillus* spp. [4,7,8].

The incidence of the disease displays an increasing trend in recent years, reflecting mainly changes in health care practices [7]. It is a well-established fact that FE is almost never observed in healthy individuals, but rather is associated with immunosuppression, intracardiac or intravascular devices, cardiac surgery and prolonged or extended-spectrum antibiotic use [3,5]. A steadily expanding population of people with compromised immunity—due to the increasing use of corticosteroids, biologics, immunosuppressants and cytotoxic drugs for malignancies, connective tissue disorders and organ-transplant recipients- has led to an increase in patients at risk for invasive fungal infection and subsequently at risk for FE [2]. With overall improvement in life expectancy, older patients are more likely to be admitted to intensive care units, have indwelling venous catheters or other procedures that predispose them to FE, such as replacement of heart valves and installation of cardiac devices [9].

Blood cultures constitute the principal method for microbiological diagnosis of FE, though their clinical reliability is diminished due to detection failures in over 50% of infected individuals [3]. *Aspergillus* endocarditis, being a notoriously delayed diagnosis, also highlights this difficulty [7]. As a consequence, serum fungal antigen detection- such as 1,3-b-D-glucan (BDG), especially for *Candida* infections, or galactomannan (GM) for *Aspergillus* infections- has emerged as a valuable adjunct in the diagnosis of fungal infections. [2] Regarding radiologic modalities, the cornerstone remains echocardiography, specifically transesophageal approach, which has higher sensitivity and specificity for IE than transthoracic echocardiography [3]. Recently, radionuclide imaging studies such as 18F-fluorodeoxyglucose (FDG) positron emission tomography (PET)/computed tomography (CT) are rapidly gaining popularity, mainly due to their ability to detect foci or septic emboli in other organs [5].

Treating FE is a highly debatable topic, especially regarding the choice of antifungal agent. Antifungal therapy active against biofilm, such as echinocandin therapy for *Candida* endocarditis or voriconazole therapy for *Aspergillus* endocarditis, is frequently the preferred choice [5,8]. Additionally, different antifungal combinations are being explored, in hope of better outcomes [9]. Early surgical intervention is highly recommended because it can prevent cardiac complications and embolic phenomena [10]. Antifungal long-term chemoprophylaxis is also gaining more ground, especially in patients who cannot undergo surgical treatment or have high risk of recurrence [2].

This review summarizes the epidemiological trends and the latest advancements in diagnosis and treatment strategies of FE in the past decade.

## 2. Methodology

An extensive literature search was performed in PubMed database and included articles published between 1 January 2015, to 31 March 2025 using the terms “fungi”, “fungal” and “endocarditis”. Articles included endocarditis guidelines, observational and retrospective cohorts, randomized control trials (when available), case series and case reports. Our aim was to present the most current evidence on FE, focusing on updates in epidemiology, diagnosis and treatment strategies.

## 3. Epidemiology

FE accounts for about 2% of overall infective endocarditis (IE) episodes [11,12]. The two most common fungi of FE are *Candida* and *Aspergillus* species. *Candida* spp. comprise 1 to 5% of all IE cases but over half of all FE cases. An increase in candidemia and in *Candida* endocarditis is expected due to rising numbers of an aging population with immunosuppression and intravascular or intracardiac devices [5,11]. Like IE, males are more likely to have *Candida* endocarditis compared to females representing 52–78% of total cases [2,11]. Despite its rarity, FE is a critical diagnosis, as it is associated with high mortality rate, ranging from 30–80% [5,11,13,14]. Among patients infected with *Candida* endocarditis, a retrospective review revealed that patients with left sided *Candida* endocarditis may have higher mortality than those with right-sided endocarditis [11]. Like bacterial IE, right-sided endocarditis is often related to intracardiac devices or a history of intravenous drug use.

Among *Candida* endocarditis, *Candida albicans* is the main cause of FE, accountable for 49.6% of FE cases and is followed by *Candida parapsilosis* (15–41%), which is the most common non-albicans *Candida* species. Next, are *C. tropicalis* (10–13%), *C.glabrata* (4–9%), *Meyeronzyma guilliermondii* (4%) and *Pichia kudriavzevil* (1%). Other species, like *Candida auris* are uncommonly encountered [2,13,14,15,16].

It is important to identify risk factors associated with the rise of FE, like prosthetic heart valves, cardiac implantable devices and injection drug use (IDU) [12,14,17]. *Candida* spp. adhere onto surfaces and form biofilms, particularly prosthetic valves. Cardiac implantable electronic devices, especially when recently placed, revised or changed (i.e., generator), are also prone to colonization and infection by fungal pathogens. Devices that have been in place for longer periods of time (more than one year) have smaller possibilities of getting infected. In a study of 70 cases of *Candida* endocarditis, 46% had a prosthetic heart valve [2].

Injection drug use (IDU) is another factor that predisposes to IE, particularly when injecting brown heroin. It is true that the IDU population has increased in several countries [14]. In a retrospective candidemia study that was conducted at the Maine Medical Centre and included 77 patients, it was shown that 16% were diagnosed with FE and from those 67% had a history of intravenous drug use [12]. In another series of 20 patients with disseminated candidiasis, tricuspid valve was the primary valve involved in those with history of IDU, with *C. albicans* being the most frequently isolated organism. *Candida parapsilosis* is also common in patients with ΙDU related FE, representing 20% of cases [16]. In addition, *C. parapsilosis* was the most commonly implicated organism between *Candida* IE cases in the Maine candidemia study [12]. Although IDU is a major risk factor for *Candinda* IE, these patients have significantly lower mortality rate than non-IDU, likely owing to young age and less comorbidities [14]. Indwelling central catheters are another risk factor for FE, while *C. albicans* has been reported to form larger and more complex biofilms than other *Candida* species [2].

*Aspergillus* accounts for 30% of FE and is more common in males [7]. An increase of *Aspergillus* endocarditis (AE) is also observed and is expected to rise due to the increased frequency of invasive procedures, cardiac device and prosthetic valve placements, together with an augmented use of immunomodulating agents. In an older cohort, *Aspergillus* accounted for 24–28% of all FE cases and between 0.25–2.5% of all IE cases reported between 1965 and 1995 [8]. According to the GAMES cohort which included 4528 consecutive patients with definite or possible IE based on the modified DUKE criteria, from 38 Spanish hospitals, conducted between January 2009 to December 2018, FE accounted for 2.2% cases, of which 10.2% were due to *Aspergillus.* In addition, 57.4% of all the AE episodes occurred in non-immunocompromised patients. In the same study, 34.4% of AE cases were seen in patients with previous valve replacement, 13.1% in patients with ICD implantation, 42.6% of them were immunosuppressed, and 63.9% of the cases occurred after surgery, probably due to direct exposure to the pathogen [3,18]. It is noteworthy that there is considerable variation in the geographic epidemiology of FE, regarding incidence and etiology. For example, in an Egyptian study, FE accounted for 11.5% of all IE cases, with *Aspergillus*, rather than *Candida*, being the predominant pathogen (8.3%) [8]. In contrast, in the Spanish GAMES cohort, *Aspergillus* was identified in only 0.2% of IE cases [18].

*Aspergillus* endocarditis has been associated with a very high mortality rate, ranging from 42 to 68%, according to a published review [4]. In another systematic review which analyzed 250 cases of FE, *Aspergillus* spp. was identified as the causative agent in 30% of cases and the overall mortality rate of AE was 40%, an independent predictor of mortality [5]. In another study that included 61 cases of AE, revealed an overall mortality rate of 52.5% [18].

Finally, endocarditis due to *Mucorales* is an exceptionally rare but life-threatening condition. It is predominantly caused by *Cunninghamella* species and occurs exclusively in immunocompromised individuals [2].

**Figure 1:**
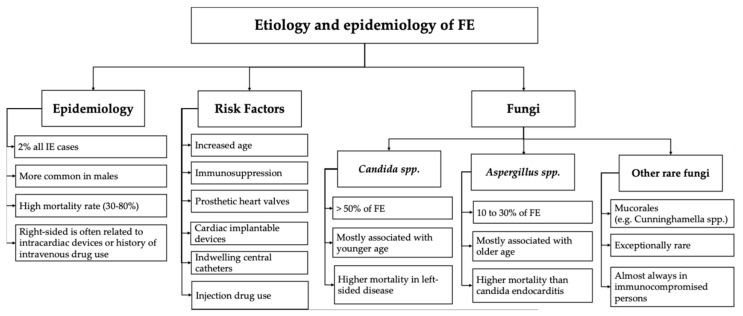
Etiology and epidemiology of FE.

In summary, the epidemiology of infectious endocarditis continues to evolve, influenced by changes in healthcare practices, an increasing prevalence of medical interventions such as the use of prosthetic valves and implantable cardiac devices as well as a rising aging population with comorbidities and/or receiving immunosuppression (Figure 1). By understanding the changes in epidemiological trends, we can guide prevention strategies and improve treatment approaches aiming to reduce morbidity and mortality associated with this critical condition.

## 4. Diagnosis

The diagnosis of fungal endocarditis represents a significant clinical challenge due to its variable clinical presentation and the poor diagnostic yield of blood cultures. In contrast to bacterial endocarditis, where culture techniques often lead to the identification of the causative organism, fungal infections frequently require a combination of microbiological, serological, molecular and imaging modalities for a definitive diagnosis. Like bacterial endocarditis, diagnosis of fungal endocarditis is based on the modified Duke criteria, which require microbiologic evidence and imaging findings suggestive of infective endocarditis [6]. However, emerging diagnostic approaches have improved the early diagnosis of fungal endocarditis.

### 4.1. Microbiologic and Molecular Methods

Blood cultures remain the cornerstone for diagnosis and should be obtained whenever a diagnosis of endocarditis is suspected [2,7,11,15]. The gold standard for the diagnosis of invasive candidiasis is a positive blood culture or culture from a normally sterile site. Despite its limitations, this method continues to be the primary means of diagnosis [8]. Apart from traditional techniques, matrix-assisted laser desorption/ionization time-of-flight (MALDI-TOF) mass spectrometry (MS) is also used in positive cultures in order to achieve a more rapid identification of the species [19]. Blood cultures tend to be positive in 21–71% of patients, a wide range probably related to suboptimal collection methods or transient fungemia [9]. Low blood culture sensitivity for *Candida* spp., complicates diagnosis and is associated with increased mortality [5]. Another important disadvantage of blood cultures is the prolonged time to positivity for *Candida* spp. compared to bacterial cultures, which may be responsible for the delay of initiation of appropriate treatment [2,15].

In cases of *Aspergillus* endocarditis, definite diagnosis may be missed due to the low yield of blood cultures for *Aspergillus*, being positive in only 4% [7,10]. This occurs probably because *Aspergillus* and other filamentous fungi have only intermittent periods of fungemia [11]. Consequently, exclusive reliance on the Duke criteria may contribute to delays in reaching a definitive diagnosis. When positive, blood cultures allow pathogen identification to the species level and susceptibility testing to be performed [2]. Definite *Aspergillus* endocarditis is defined when pathological criteria are fulfilled, by the presence of *Aspergillus* in culture, molecular techniques or histological examination of a vegetation, or a vegetation that has embolised or an intracardiac abscess specimen or other pathological lesions [12,13,20]. This approach is also emphasized by the European Organisation for Reseasrch and Treatment of Cancer/Mycoses Study Group (EORTC/MSG) Criteria [21]. In practice, delayed diagnosis is typical, with a significant proportion of cases identified post-mortem [7,8].

An evolving molecular method for pathogen identification is next generation sequencing (NGS). Metagenomic NGS (mNGS) is useful in detecting microbial cell-free DNA (mcfDNA) in plasma allowing earlier detection and diagnosis of fungal infections when conventional serum biomarkers yield limited results [21]. Multiplex PCR is performed by testing the given sample against a predetermined panel of pathogen targets, however missing out on the causative pathogen, if it is absent from the panel. NGS has the ability to compare nucleic acid sequences found in the sample to an extensive database of pathogens and hence may be helpful in facilitating earlier diagnosis. The role of NGS in identification of fungal pathogens like *Aspergillus* is under evaluation and process standardization is needed [7,22].

Galactomannan (GM) and β-D-glucan (BDG) assays represent non-culture based diagnostic tools that aid in the detection of invasive fungal infections, including fungal endocarditis. BDG, a component of the fungal cell wall, is present in various fungi such as *Candida* and *Aspergillus* species, and its augmented levels can support the diagnosis of fungal endocarditis, particularly in the presence of negative blood cultures. For example, a case study demonstrated the utility of BDG assay in the early detection of fungal endocarditis in a patient, leading to prompt antifungal therapy initiation [14]. GM, a polysaccharide component of *Aspergillus* species, can be detected in serum and other body fluids, but their role in FE is less well-defined due to limitations in the sensitivity and specificity in this context. However, the combination of GM and BDG assays may enhance diagnostic accuracy for invasive fungal infections [15].

If surgery is performed, histopathological examination with adjunctive microbiological and molecular-based testing (pan-fungal or *Candida*-specific PCR) can lead to confirmation of the pathogenic microorganism in case of blood culture negative endocarditis and indeterminate diagnosis. Tissue samples should not be placed in formalin until the appropriate portions have been sent to the microbiology laboratory [2].

### 4.2. Imaging

**Figure 2:**
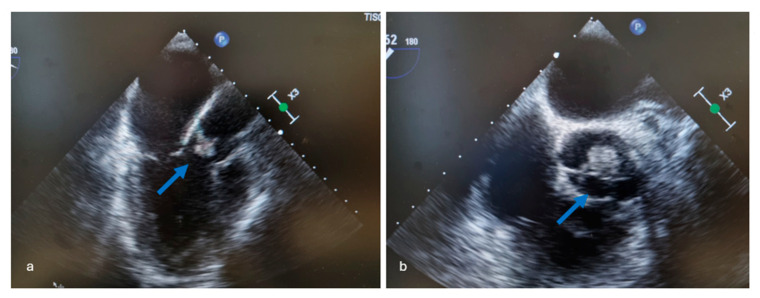
Echocardiographic images of a large vegetation (arrows) due to *Candida* spp. aortic valve endocarditis (**a**,**b**).

Echocardiography is recommended in patients with persistent candidemia to assess for fungal endocarditis [22]. Usually a transthoracic echocardiography (TTE) is performed first, and then a TEE is considered according to the TTE findings, although when suspicion of IE is high one can proceed directly to the TEE [19]. Although transthoracic echocardiography (TTE) is widely available and relatively rapid, its sensitivity to adequately evaluate all valves is often limited, especially in the presence of prosthetic valves or intracardiac devices and in obese patients. In these cases, TEE is the first-line imaging technique. The reported sensitivity of TTE for infective endocarditis (IE) is 70% for native valves and 50% for prosthetic valve endocarditis, while the sensitivity of TEE is 96% for native valves and 92% for prosthetic valves [2]. A retrospective study conducted in a tertiary hospital that recorded 263 cases of candidemia in adult patients, revealed a prevalence of 11.5% *Candida* endocarditis (CIE) cases among patients with candidemia studied with TEE. Based on these findings, systematic echocardiography is justified in this patient population, since the presence of a valvular prosthesis was the sole significant distinguishing factor between individuals with and without *Candida* IE [19]. While echocardiography does not allow *Candida* endocarditis to be distinguished from endocarditis due to other pathogens, fungal endocarditis lesions are often large and highly mobile. Data from the MYCENDO study showed vegetations of 13 mm in half of the 30 cases, with vegetation size ranging from 4 to 30 mm, while another report of 15 cases also showed large vegetations in *C. albicans* endocarditis with a mean size of 19.4 mm (range: 8.8 to 29.9 mm). Hyperechoic lesions are also suggestive of vegetations caused by *Candida* spp. [2]. In Figure 2, an echocardiographic image of a large vegetation due to *Candida* aortic valve endocarditis is depicted. While European guidelines recommend routine screening for endocarditis by echocardiography and frequent physical examination in patients diagnosed with candidemia, such recommendation is lacking in current Infectious Diseases Society of America (IDSA) guidelines due to the relatively low prevalence (1.9% to 5.9%) of *Candida* endocarditis in patients with candidemia [2].

Other imaging modalities to evaluate IE include cardiac computed tomography (CCT), F-Fluorodeoxyglucose-Positron Emission Tomography/Computed Tomography (FDG-PET/CT), and Indium-111 Leukocyte-Scintigraphy. CCT has shown promise when it comes to diagnosing and detecting complications of IE from other pathogens, though it has not yet been evaluated in *Candida* endocarditis. Moreover, CCT might provide additional information of anatomical details, which could be useful in surgical planning. There have been reports of successful diagnosis of *Candida* endocarditis with CCT [2].

The role of PET/CT in the diagnostic process of FE has also been examined. 18F PET/CT was included in the 2015 ESC IE diagnostic criteria for prosthetic valve endocarditis and is particularly useful when echocardiography fails to confirm the diagnosis despite high clinical suspicion. The 2023 Duke-ISCVID IE criteria have included 18F FDG PET/CT as an imaging modality for diagnosing IE, especially in cases of prosthetic material. In native valves, PET/CT is not sensitive in excluding IE but it has a very high positive predictive value if the uptake in the valve is high [7]. A retrospective, single-center analysis of PET/CT sensitivity in CE was performed in 14 cases of *Candida* endocarditis at Regensburg University Hospital. The findings revealed that the sensitivity of ^18F-FDG PET/CT was 57.1%, comparable to the 54.5% sensitivity of transthoracic echocardiography (TTE). Despite the modest sensitivity, the study highlighted the potential advantages of PET/CT in detecting extracardiac infectious foci or septic emboli, which can be crucial in confirming the diagnosis of *Candida* endocarditis, especially in cases with diagnostic uncertainty [5]. While the sensitivity of FDG-PET is low for native valve endocarditis, with a pooled sensitivity of 31%, its diagnostic accuracy improves in cases of prosthetic valves and intracardiac devices, where imaging via echocardiography has limitations. Moreover, FDG-PET may facilitate early visualization of infection in cases with negative initial echocardiograms and can also help identify systemic emboli [2]. In a recent study, sensitivity of PET/CT was not compromised in patients with febrile neutropenia, but data on PET/CT in patients with invasive fungal disease and neutropenia are limited [23].

In conclusion, diagnosis is challenging due to the often-subacute presentation and difficulty in isolating fungi from blood cultures. Diagnosis must be suspected in the appropriate context [14]. Clinical suspicion should be high in immunocompromised patients, intravenous drug users, those with prosthetic valves, or prolonged hospitalization with central venous catheters. Diagnosis relies on a combination of clinical features, imaging (especially transesophageal echocardiography) and microbiological or histopathologic evidence from blood, tissue or embolic material. Due to its high morbidity and mortality, prompt diagnosis is critical for improved outcomes.

## 5. Treatment of Fungal Endocarditis

Fungal endocarditis (FE) is a life-threatening condition with mortality rates exceeding 50%, creating the need for multidisciplinary therapeutic strategies [16]. While effective management remains challenging due to limited prospective data, the complexity of the disease, and the diverse nature of the pathogens involved, clinicians have made significant advancements in recent years.

### 5.1. Surgical Management

Surgical intervention is a critical component in managing fungal endocarditis across *Candida*, *Aspergillus*, and non-*Candida* non-*Aspergillus* species. In general, earlier surgical intervention is favored in fungal endocarditis compared to bacterial endocarditis due to the high mortality rates associated with medical therapy alone [17]. As is the case with bacterial infective endocarditis, indications for surgery in FE include severe heart failure, prosthetic valve involvement, invasion beyond the valve leaflets, the presence of large mobile vegetations (>10 mm), embolic events, or hemodynamic instability, with early surgery recommended to improve outcomes [24] (Table 1).

#### 5.1.1. Surgical Treatment in *Candida* Endocarditis

For *Candida* spp. endocarditis, early valve replacement or vegetectomy combined with systemic antifungals (e.g., amphotericin B or echinocandins) is strongly recommended [18,32]. The impact of surgical intervention on mortality in *Candida* endocarditis remains debated, with conflicting evidence across the available studies (Table 2). Some reviews, such as a meta-analysis by Ellis et al. (1965–1985), suggested a trend toward improved survival with combined antifungal therapy and surgery (55% 1-year survival) compared to antifungal therapy alone (36%) [20]. Similarly, Steinbach et al.’s analysis of 163 *Candida* endocarditis cases (1996–2002) reported lower odds of death with surgery (prevalence odds ratio: 0.56; 95% CI: 0.16–1.99) [33].

However, contemporary prospective studies challenge these findings. Arnold et al. (2015) observed no mortality difference between surgical and medical management in 70 *Candida* endocarditis cases (in-hospital mortality: 38% vs. 34%; 1-year mortality: 66% vs. 62%) [13]. Baddley et al. (2008) similarly found no survival benefit with surgery in 33 patients (30.3% vs. 27.8% mortality) [27]. The ESCAPE study (2020) reported lower in-hospital mortality with surgery in prosthetic valve endocarditis (27% vs. 64%), but long-term survival did not differ, suggesting surgery’s benefits may be limited to acute-phase complications [28]. Notably, surgery was more common in younger, healthier patients, whereas older individuals with comorbidities often received medical therapy alone, complicating direct comparisons.

#### 5.1.2. Surgical Treatment in *Aspergillus* Endocarditis

*Aspergillus* spp. endocarditis presents its own unique set of challenges. Aggressive surgical resection of infected tissue and valve replacement is nearly mandatory due to the pathogen’s propensity for large vegetations and systemic embolization [22,35]. The Infectious Diseases Society of America (IDSA) 2016 guidelines strongly advocate for early surgical intervention combined with systemic antifungal therapy—typically voriconazole or lipid formulations of amphotericin B—to mitigate embolic complications and valvular decompensation, though lifelong antifungal suppression is recommended post-surgery due to high recurrence risks (30%) despite limited evidence supporting this practice [36]. Observational studies further emphasize the importance of surgery: Kalokhe et al. demonstrated a stark survival disparity, with only 4% survival (2/53) in patients managed medically versus 32% survival (17/53) when surgery was combined with antifungals [37]. Similarly, McCormack et al. reported 88% survival (7/8) in patients undergoing valve replacement compared to 6% survival (1/17) with conservative treatment alone, highlighting surgery’s life-saving potential [35]. Reischies & Hoenigl (2014) note that historical data suggested survival benefits with surgery, but modern cohorts show equivalent outcomes due to improved antifungals and patient-specific factors, such as comorbidities influencing mortality more than treatment modality [38]. Embolic events, occurring in up to 70% of cases, drive the urgency for surgical removal of vegetations, particularly in prosthetic valve infections or pacemaker wire involvement, where thoracotomy is preferred for large vegetations to avoid fatal intraprocedural emboli [21,38]. Despite combined surgical and antifungal therapy (e.g., voriconazole ± echinocandins), mortality remains high, with relapses occurring in 30–40% of survivors, creating a need for long-term suppressive therapy [39].

#### 5.1.3. Surgical Treatment in Non-*Candida* and Non-*Aspergillus* Endocarditis

For non-*Candida* and non-*Aspergillus* fungal endocarditis, surgical treatment is similarly unavoidable due to the limited effectiveness of medical therapy alone. Rare fungal pathogens often form large vegetations that are resistant to antifungal penetration, making surgical removal necessary [40]. Valve replacement or vegetectomy is typically performed in conjunction with prolonged antifungal therapy tailored to the pathogen’s susceptibility profile. Early surgery is particularly critical in cases involving hemodynamic instability, progressive vegetation growth, or recurrent embolization [41]. Across all fungal species, a multidisciplinary approach integrating surgical and medical therapies remains the standard for improving patient outcomes.

### 5.2. Medical Management

#### 5.2.1. Antifungal Therapeutic Options for *Candida* spp. Endocarditis

For *Candida* endocarditis affecting native or prosthetic valves, current guidelines recommend initial treatment with lipid formulations of amphotericin B (L-AmB) with or without flucytosine, or alternatively, high-dose echinocandins [32,42,43]. L-AmB is typically administered at doses of 3–5 mg/kg/day and is often combined with flucytosine (25 mg/kg four times daily) to enhance fungal clearance and synergistically target biofilms [36]. L-AmB exerts rapid, concentration-dependent fungicidal activity via ergosterol binding but requires vigilant renal and electrolyte monitoring. As such, clinicians must carefully balance its nephrotoxicity risks against its therapeutic benefits. Reduced activity against biofilms, which are a hallmark of *Candida* infections, further limits its utility as monotherapy [36,44].

In contrast, recent studies have highlighted the increasing role of echinocandins, such as caspofungin, micafungin, and anidulafungin, in the treatment of *Candida* endocarditis [13,43,44]. Echinocandins inhibit 1,3-β-D-glucan synthase and are fungicidal against most *Candida* species, with strong biofilm activity and favorable safety; however, activity can be reduced against *Candida parapsilosis* complex, necessitating close attention to MICs [2,44]. These agents offer a double advantage: superior biofilm penetration and a more favorable safety profile compared to L-AmB. High-dose echinocandin regimens are now endorsed as first-line therapy in many cases, either as monotherapy or in combination with fluconazole, flucytosine, or amphotericin B, depending on drug availability [24,44].

Having said that, the choice between L-AmB and echinocandins depends on patient-specific factors (e.g., renal/hepatic function), fungal species susceptibility (e.g., *C. parapsilosis* resistance to echinocandins), and the severity of the patient’s condition. Combination regimens, albeit promising, lack proper validation from randomized controlled trials and remain largely supported by observational data [13]. International guidelines strongly recommend minimum treatment duration of six weeks following surgical intervention for fungal endocarditis, with extended courses suggested in the presence of complications, such as paravalvular abscesses. In cases where surgical management is not possible—often due to comorbidities or patient refusal— prolonged antifungal therapy is required to ensure adequate infection control and reduce the risk of recurrence [36]. Step-down therapy to azoles like fluconazole (400–800 mg/day) or voriconazole (4 mg/kg twice daily) is commonly employed after 2–4 weeks of induction therapy for azole-susceptible isolates, once the patient is clinically stable and bloodstream cultures return negative [5,12,13] Therapeutic drug monitoring is advisable for voriconazole and posaconazole when used for step-down or suppression (typical trough targets ≥ 1 μg/mL; voriconazole 1–5.5 μg/mL), particularly in hepatic dysfunction, significant drug–drug interactions or variable absorption [13,24] (Table 3).

#### 5.2.2. Antifungal Therapeutic Options for *Aspergillus* spp. Endocarditis

The medical management of *Aspergillus* endocarditis (AE) alone presents significant challenges, due to its aggressive nature and inherent resistance to antifungal therapy. Survival rates remain alarmingly low, with only 4% of cases achieving cure compared to 32% survival when combined with surgery [37].

Current guidelines recommend voriconazole as first-line treatment, administered intravenously at a loading dose of 6 mg/kg twice daily for two doses, followed by a maintenance dose of 4 mg/kg twice daily [32,42]. Voriconazole is preferred due to its superior penetration into tissues, including the central nervous system, and its proven efficacy against invasive aspergillosis [36]. Therapeutic drug monitoring (TDM) is critical, ensuring voriconazole trough concentrations remain between 1–5.5 μg/mL to optimize effectiveness while minimizing toxicity risks such as hepatotoxicity and visual disturbances [32].

L-AmB, dosed at 3–5 mg/kg/day, serves as an alternative in cases of azole resistance or voriconazole intolerance. However, clinicians must weigh its limited biofilm penetration against its higher nephrotoxicity risks [32].

Combination therapy involving voriconazole with an echinocandin (e.g., caspofungin at a loading dose of 70 mg followed by 50 mg daily) may offer improved outcomes in refractory cases; observational data suggest increased survival rates compared to monotherapy, though randomized trials validating this approach are lacking [45,46].

Alternative treatment options for *Aspergillus* endocarditis have emerged in recent years, offering potential benefits in cases of drug resistance, intolerance, or treatment failure. Isavuconazole, a second-generation triazole, has demonstrated efficacy comparable to voriconazole with a potentially improved safety profile and fewer drug interactions [47]. Posaconazole, another broad-spectrum triazole, has shown similar efficacy to voriconazole but may have a higher incidence of hepatobiliary and renal adverse events [46].

It is crucial to note that antifungal therapy alone is rarely sufficient for *Aspergillus* endocarditis, and surgical intervention is typically necessary in conjunction with prolonged antifungal treatment to improve survival outcomes [38]. Following surgery, antifungal therapy should be continued for a minimum of 12 weeks, with lifelong suppressive therapy using voriconazole or posaconazole recommended for patients with prosthetic valves or persistent immunosuppression [32,42]. Despite these aggressive measures, AE continues to carry significant morbidity and mortality, emphasizing the need for prompt diagnosis and multidisciplinary management (Table 4).

#### 5.2.3. Treatment of Fungal Endocarditis Caused by Non-*Candida*, Non-*Aspergillus* Species

Treatment options for fungal endocarditis caused by non-*Candida* and non-*Aspergillus* species are less well-defined due to the rarity of these infections and limited clinical data. Nevertheless, therapy hinges on susceptibility testing, antifungal treatment tailored to the pathogen involved, and surgical intervention where necessary [43]. Unlike *Candida* species, rare non-*Candida* yeasts tend to display higher minimum inhibitory concentrations (MICs) against echinocandins, making this class of antifungals less effective and generally not recommended for treatment. Amphotericin B formulations are typically considered the first-line agents for these infections; however, exceptions exist, such as *Trichosporon* infections, where azole antifungals are preferred due to their superior efficacy against this pathogen [48].

For species such as *Trichosporon*, voriconazole has demonstrated the best results in case series, although some subtypes exhibit resistance to amphotericin B [41]. For other rare fungi, such as *Fusarium* or *Scedosporium*, voriconazole or posaconazole are often employed due to their broad-spectrum activity [49,50]. Surgical intervention, including valve replacement, is strongly recommended alongside antifungal therapy, as isolated medical treatment is typically insufficient to eradicate these infections [51]. Extended courses of systemic antifungal therapy, often lasting six weeks or more, are required, with long-term suppressive therapy considered in cases involving prosthetic valves or high relapse risk. Ultimately, a multidisciplinary approach remains critical to optimize outcomes in these challenging cases [32].

#### 5.2.4. Newer Antifungals and Their Role in FUNGAL Endocarditis Treatment

Newer antifungals such as rezafungin, ibrexafungerp, olorofim and fosmanogepix are being studied as potential future treatment options, each with distinct mechanisms and potential roles in salvage therapy [52,53,54]. Rezafungin is a next-generation echinocandin antifungal agent that inhibits 1,3-β-D-glucan synthase in fungal cell walls, demonstrating concentration-dependent fungicidal activity against *Candida* species with a markedly prolonged half-life (>130 h) that enables once-weekly intravenous dosing [55,56]. While FDA-approved for candidemia and invasive candidiasis in adults with limited alternatives, rezafungin has not been formally studied in endocarditis [57,58]. Emerging case reports describe successful use weekly rezafungin as consolidation or suppressive therapy in both native and prosthetic valve *Candida* endocarditis, including *C. glabrata* and *C. parapsilosis* cases [59,60,61]. One article documented 8 weeks of outpatient weekly infusions and data from an expanded access program reported treatment durations of 5–39 weeks (some up to 12 months) with generally good tolerability and culture clearance, though outcomes varied due to underlying comorbidities [62]. Given its once-weekly pharmacokinetics, potent antibiofilm activity and favorable tolerability, rezafungin could be considered in patients with *Candida* endocarditis and limited treatment options [55,56,58]. However, its use should be individualized within a multidisciplinary endocarditis team approach, because current evidence is limited to case reports and small observational experiences [52,63].

Ibrexafungerp, is a first-in-class triterpenoid glucan synthase inhibitor, which retains oral biofilm activity and partial non-overlapping resistance with echinocandins, offering theoretical advantages for *Candida* endocarditis [64]. Clinical evidence for ibrexafungerp in fungal endocarditis is limited to isolated cases embedded within compassionate-use and observational programs for invasive candidiasis (notably FURI and CARES), lacking endocarditis-specific data from randomized trials or prospective cohorts. Reports describe its use mainly as oral salvage or step-down therapy in refractory *Candida* infections—including rare endovascular/endocarditis cases —when standard options were precluded by resistance or intolerance [65,66].

Olorofim (an orotomide) inhibits fungal dihydroorotate dehydrogenase and demonstrates potent activity against *Aspergillus* spp., including azole-resistant strains, with oral bioavailability and preliminary CNS penetration data [67]. A recently published phase 2b trial evaluated its efficacy and safety in 202 patients with invasive fungal infections and demonstrated a 28.7% global response rate at day 42 and acceptable tolerability over 84 days. Olorofim’s potent activity against *Aspergillus*, *Lomentospora*, and *Scedosporium* suggests it could serve as salvage therapy in rare cases of endocarditis when standard agents fail and surgery is contraindicated, although no endocarditis cases were included in the trial [68]. Given its lack of activity against *Candida*, olorofim’s role in fungal endocarditis remains investigational and should be limited to highly individualized use, only under expert oversight [69].

Fosmanogepix inhibits the fungal enzyme Gwt1 and has been shown to be effective against a broad spectrum of fungi, including yeasts and moulds such as *Candida, Aspergillus*, and *Fusarium,* Although, there is limited clinical evidence to date, its potent antibiofilm effect makes it a likely option for the treatment of fungal endocarditis (54).

### 5.3. Suppressive Therapy

Chronic suppressive therapy is a critical component of managing fungal endocarditis, particularly in high-risk scenarios such as prosthetic valve infections or non-surgical candidates. The available guidelines emphasize that suppressive therapy decisions should be individualized based on patient-specific factors, including the presence of prosthetic devices, fungal species involved, and comorbidities. While fluconazole remains the most widely used agent, other azoles such as posaconazole and isavuconazole may be considered in cases of resistance or intolerance, though evidence for their use remains limited [12,36]. For native valve endocarditis, the IDSA 2016 guidelines recommend step-down oral fluconazole (400–800 mg daily) after initial treatment with amphotericin B or echinocandins, provided the isolate is susceptible and blood cultures have cleared [36]. For fungal pathogens like *Candida parapsilosis*, long-term suppressive therapy has been shown to enable prolonged symptom-free survival in patients who are not surgical candidates [33]. However, evidence supporting lifelong suppression in native valve cases remains limited, with guidelines emphasizing individualized decisions based on clinical stability and susceptibility testing. In prosthetic valve endocarditis, chronic suppression is more strongly advised. Arnold et al. observed that 64% of *Candida* endocarditis patients received lifelong fluconazole post-surgery, particularly for recurrent or complicated cases [13]. Similarly, Dhakal et al. and Melgar et al. highlighted the necessity of indefinite fluconazole (400–800 mg daily) to prevent relapse in prosthetic valve infections, given the high recurrence risk (30%) and biofilm persistence [70,71]. Ioannou et al. reinforced this approach, noting reduced relapse rates with prolonged azole therapy in prosthetic cases [72]. For non-surgical candidates, Smego et al. and the IDSA guidelines recommend indefinite fluconazole suppression if valve replacement is not feasible, despite limited direct evidence [36,73]. This strategy is supported by case series showing stabilization in medically managed patients, though resistance and adherence challenges persist. Overall, suppressive therapy hinges on isolate susceptibility, patient tolerance, and surgical feasibility, with fluconazole as the cornerstone agent. While data are predominantly observational, these approaches aim to mitigate the high mortality (36–59%) and recurrence risks inherent to fungal endocarditis [36,43,45]

Initiating lifelong suppressive therapy in fungal endocarditis is an inherently complex clinical decision that requires balancing potential benefits against substantial risks, notably antifungal resistance and cumulative long-term toxicity [4]. While the evidence supporting indefinite suppression derives primarily from observational studies and case series rather than randomized controlled trials—a limitation reflecting both the rarity of fungal endocarditis and ethical challenges of withholding potentially life-saving therapy—several significant concerns warrant consideration. Prolonged antifungal exposure poses multifaceted risks, including the emergence of resistance through target site mutations, upregulation of efflux pumps, and biofilm-mediated tolerance mechanisms. Additionally, cumulative toxicity from chronic azole therapy presents notable concerns, particularly hepatotoxicity, significant drug-drug interactions, and the theoretical malignancy risk associated with long-term voriconazole use [4,22]. The risk-benefit assessment must therefore consider individual patient factors including prosthetic material type (mechanical versus bioprosthetic valves), immune status, surgical candidacy, pathogen-specific resistance patterns, overall performance status and patient preferences.

## 6. Conclusions and Key Messages

Epidemiology and risk factors: Fungal Endocarditis is rare but lethal, with mortality rates exceeding 50%. *Candida* species cause over half of FE cases, followed by *Aspergillus.* Key risk factors include prosthetic heart valves, intracardiac devices, immunosuppression, intravenous drug use, and prolonged hospitalisation with invasive catheters. Males are more frequently affected, and outcomes are poorest in *Aspergillus* infections.

Diagnostic Challenges: Blood cultures, the traditional diagnostic method, have limited sensitivity. Serum biomarkers (β-D-glucan, galactomannan) improve early detection, especially in culture-negative cases. Advanced imaging modalities (particularly 18F-FDG-PET/CT) are increasingly used to guide the diagnostic process.

Emerging Tools and Strategies: Metagenomic next-generation sequencing (mNGS) shows promise for detecting microbial cell-free DNA in plasma, enabling earlier diagnosis. Histopathology and PCR of excised tissue remain gold standards in establishing a definitive diagnosis.

Clinical Awareness: FE should be suspected in high-risk patients, even with non-specific symptoms (e.g., prolonged fever, embolic events). Transesophageal echocardiography vastly outperforms transthoracic methods of detecting vegetations. Timely diagnosis and tailored therapy are crucial in mitigating this condition’s devastating prognosis.

Management: The management of Fungal Endocarditis requires a coordinated multidisciplinary approach combining appropriate antifungal therapy and timely surgical intervention. The choice and duration of antifungal treatment should be guided by the causative organism, susceptibility patterns, and patient factors, with lifelong suppressive therapy often necessary for prosthetic valve infections. Surgical intervention remains essential in most cases, particularly with hemodynamic compromise, large vegetations, or persistent fungemia despite medical therapy.

Future Challenges: FE demands a high index of clinical suspicion, rapid multimodal diagnostics and aggressive treatment. Despite these interventions, several questions remain unresolved, including the optimal timing of surgery, efficacy of combination antifungal regimens, management of antifungal resistance, and the appropriate duration of suppressive therapy. The rarity of fungal endocarditis limits high-quality evidence, highlighting the need for multicenter collaborations to develop standardized protocols and evaluate emerging antifungals. Until then, individualized care delivered by multidisciplinary endocarditis teams remains the cornerstone of management for this challenging condition with persistently high mortality.

## Figures and Tables

**Table 1:** Data on the percent of patients with FE, subjected to surgical intervention.

Study/(N)	Year	Surgery	Reference
Siciliano, R.F., et al. (78)	1980–2015	59%	[11]
Sankar, N.P., et al. (12)	2013–2018	58%	[12]
Arnold, C.J., et al. (70)	2000–2010	39%	[13]
Huggins, J.P., et al. (703)	2015–2019	22.5%	[25]
Lefort, A., et al. (30)	2005–2007	43%	[26]
Baddley, J.W., et al. (33)	2000–2005	45.5%	[27]
Rivoisy, C., et al. (46)	2001–2005	41%	[28]
Giuliano, S., et al. (140)	1997–2014	55%	[29]
Pierrotti, L., et al. (152)	1995–2000	78/119 (65.5%) No data for 33 patients.	[30]
Ellis, M.E., et al. (270)	1965–1995	41%	[31]

**Table 2:** Impact of Surgery in *Candida* Endocarditis.

Study/(N)	Combination Therapy (Surgery + Antifungal)	Antifungal Therapy	Endpoint	*p* Value	Reference
Arnold, C.J., et al. (70)	38%	34%	In hospital mortality	NS (0.77)	[13]
Rivoisy, C., et al. (46)	50%	68%	1 year mortality	NS	[28]
Ellis, M.E., et al. (270)	45%	64%	1 year mortality	<0.05	[31]
Steinbach, W.J., et al. (163)	65.6%	34.4%	In hospital mortality	OR (0.56; CI: 0.16 to 1.99) NS	[33]
Meena, D.S., et al. (250)	64.8%	35.2%	In hospital mortality	HR (0.20; CI: 0.09–0.42) <0.01	[34]

**Table 3:** Antifungal Dosing Chart in *Candida* Endocarditis.

Agent	Dose	Key Notes
Liposomal Amphotericin B	3–5 mg/kg/day	Combine with flucytosine for synergy
Caspofungin	150 mg/day	Echinocandins are preferred for their increased biofilm activity
Micafungin	150 mg/day	
Anidulafungin	200 mg/day	
Fluconazole	400–800 mg/day	Step-down for susceptible isolates—Chronic suppressive therapy
Voriconazole	3–4 mg/kg 2x daily	May be utilized for isolates demonstrating fluconazole resistance
Posaconazole	300 mg daily, delayed release tablets	May be utilized for isolates demonstrating fluconazole resistance
Flucytosine	25 mg/kg 4x daily	Always combine with L-AmB/echinocandin

**Table 4:** Antifungal Dosing Chart in *Aspergillus* Endocarditis.

Antifungal Agent	Dosing Regimen	TDM and Target Trough Concentrations	Adverse Events
Voriconazole	IV: 6 mg/kg twice daily (loading dose for 2 doses), then 4 mg/kg twice daily Oral: 200–300 mg twice daily	1–5.5 µg/mL	Hepatotoxicity, rash, visual disturbances, hallucinations, photosensitivity, periostitis
Liposomal Amphotericin B	3–5 mg/kg/day	Not indicated	Nephrotoxicity, infusion reactions, electrolyte imbalances (hypokalemia, hypomagnesemia)
Isavuconazole	372 mg every 8 h for 6 doses (loading), then 372 mg once daily (maintenance dose)	>1 µg/mL	Edema, hypokalemia, abdominal pain, hepatotoxicity
Posaconazole	300 mg twice daily for 2 doses (loading), then 300 mg once daily (maintenance dose)	>1 µg/mL	Gastrointestinal upset, hypokalemia, hypertension
Caspofungin	70 mg loading dose on day 1, then 50 mg once daily	Not indicated	Hepatotoxicity, infusion reactions
Micafungin	100–150 mg once daily	Not indicated	Gastrointestinal upset, infusion reactions
Anidulafungin	200 mg loading dose on day 1, then 100 mg once daily	Not indicated	Hypokalemia, diarrhea, infusion reactions

## References

[1] Li M., Kim J.B., Sastry B.K.S., Chen M. (2024). Infective endocarditis. Lancet.

[2] Thompson G.R., Jenks J.D., Baddley J.W., Lewis J.S., Egger M., Schwartz I.S., Boyer J., Patterson T.F., Chen S.C.-A., Pappas P.G. (2023). Fungal Endocarditis: Pathophysiology, Epidemiology, Clinical Presentation, Diagnosis, and Management. Clin. Microbiol. Rev..

[3] Ammannaya G.K.K., Sripad N. (2019). Fungal endocarditis: What do we know in 2019?. Pol. Heart J..

[4] Naguthevar S., Ravindra A., Kumar D., Meena D.S., Bohra G.K., Jain V., Garg M.K., Deora S., Choudhary R. (2024). Emerging trends in fungal endocarditis: Clinical complexity, diagnostic challenges, and therapeutic implications–a case series and literature review. Ther. Adv. Infect. Dis..

[5] Hitzenbichler F., Joha T., Simon M., Grosse J., Menhart K., Hellwig D., Camboni D., Sag S., Sag C.M., Hanses F. (2020). Candida Endocarditis in Patients with Candidemia: A Single-Center Experience of 14 Cases. Mycopathologia.

[6] Yuan S.-M. (2016). Fungal endocarditis. Braz. J. Cardiovasc. Surg..

[7] Gopal K., Bhaskaran P.N., Moni M., Shashindran N. (2024). Aspergillus endocarditis. Indian Heart J..

[8] Meshaal M.S., Labib D., Said K., Hosny M., Hassan M., Al Aziz S.A., Elkholy A., Anani M., Rizk H., Ballotta A. (2018). Aspergillus endocarditis: Diagnostic criteria and predictors of outcome, A retrospective cohort study. PLoS ONE.

[9] Sahra S., Javed A., Jahangir A., Thind S.K. (2023). Pharmacological options for Candida albicans Endocarditis at the roadblock with irrecoverable prosthetics and drug interactions: A case report and review of literature. BMC Infect. Dis..

[10] Correia J.L., Fiuza J.G., Ferreira G., Almeida M.D., Moreira D., Neto V.D. (2024). Embolic stroke and misidentification candida species endocarditis: Case presentation and literature review. Diagn. Microbiol. Infect. Dis..

[11] Siciliano R.F., Gualandro D.M., Sejas O.N.E., Ignoto B.G., Caramelli B., Mansur A.J., Sampaio R.O., Pierrotti L.C., Barbosa G., Golebiovski W. (2018). Outcomes in patients with fungal endocarditis: A multicenter observational cohort study. Int. J. Infect. Dis..

[12] Sankar N.P., Thakarar K., Rokas K.E. (2020). Candida infective endocarditis during the infectious diseases and substance use disorder syndemic: A six-year case series. Open Forum Infect. Dis..

[13] Arnold C.J., Johnson M., Bayer A.S., Bradley S., Giannitsioti E., Miró J.M., Tornos P., Tattevin P., Strahilevitz J., Spelman D. (2015). Candida Infective Endocarditis: An Observational Cohort Study with a Focus on Therapy. Antimicrob. Agents Chemother..

[14] Morelli M.K., Veve M.P., Lorson W., Shorman M.A. (2021). *Candida* spp. infective endocarditis: Characteristics and outcomes of twenty patients with a focus on injection drug use as a predisposing risk factor. Mycoses.

[15] Vaideeswar P. (2015). Candidial Endocarditis: A Single-Institute Pathological Analysis. Mycopathologia.

[16] Guo P., He Y., Fan R., Wu Z., Chen Y., Huang Y., Liao K., Chen P. (2021). A case series of medically managed Candida parapsilosis complex prosthetic valve endocarditis. Ann. Clin. Microbiol. Antimicrob..

[17] Shin S.U., Yu Y.H., Kim S.S., Oh T.H., Kim S.E., Kim U.J., Kang S.-J., Jang H.-C., Park K.-H., Jung S.I. (2020). Clinical characteristics and risk factors for complications of candidaemia in adults: Focus on endophthalmitis, endocarditis, and osteoarticular infections. Int. J. Infect. Dis..

[18] Valerio M., Camici M., Machado M., Galar A., Olmedo M., Sousa D., Antorrena-Miranda I., Fariñas M.C., Hidalgo-Tenorio C., Montejo M. (2022). *Aspergillus* endocarditis in the recent years, report of cases of a multicentric national cohort and literature review. Mycoses.

[19] Fernández-Cruz A., Cruz Menárguez M., Muñoz P., Pedromingo M., Peláez T., Solís J., Rodríguez-Créixems M., Bouza E., on behalf of the GAME Study Group (Grupo de Apoyo al Manejo de la Endocarditis) (2015). The search for endocarditis in patients with candidemia: A systematic recommendation for echocardiography? A prospective cohort. Eur. J. Clin. Microbiol. Infect. Dis..

[20] Chevalier K., Barde F., Benhamida S., Le Meur M., Thyrault M., Bentoumi Y., Lau N., Lebut J. (2021). Invasive aspergillosis and endocarditis. Rev. Med. Interne.

[21] Peter Donnelly J., Chen S.C., Kauffman C.A., Steinbach W.J., Baddley J.W., Verweij P.E., Clancy C.J., Wingard J.R., Lockhart S.R., Groll A.H. (2020). Revision and update of the consensus definitions of invasive fungal disease from the european organization for research and treatment of cancer and the mycoses study group education and research consortium. Clin. Infect. Dis..

[22] Bays D.J., Jenkins E.N., Lyman M., Chiller T., Strong N., Ostrosky-Zeichner L., Hoenigl M., Pappas P.G., Thompson G.R. (2024). Epidemiology of Invasive Candidiasis. Clin. Epidemiol..

[23] El Zakhem A., Istambouli R., Jabbour J.F., Hindy J.R., Gharamti A., Kanj S.S. (2022). Diagnosis and Management of Invasive Candida Infections in Critically Ill Patients. Semin. Respir. Crit. Care Med..

[24] Chandra J., Kuhn D.M., Mukherjee P.K., Hoyer L.L., McCormick T., Ghannoum M.A. (2001). Biofilm formation by the fungal pathogen *Candida albicans*: Development, architecture, and drug resistance. J. Bacteriol..

[25] Huggins J.P., Hohmann S., David M.Z. (2021). *Candida* Infective Endocarditis: A Retrospective Study of Patient Characteristics and Risk Factors for Death in 703 United States Cases, 2015–2019. Open Forum Infect. Dis..

[26] Lefort A., Chartier L., Sendid B., Wolff M., Mainardi J.-L., Podglajen I., Desnos-Ollivier M., Fontanet A., Bretagne S., Lortholary O. (2012). Diagnosis, management and outcome of Candida endocarditis. Clin. Microbiol. Infect..

[27] Baddley J.W., Benjamin D.K., Patel M., Miró J., Athan E., Barsic B., Bouza E., Clara L., Elliott T., Kanafani Z. (2008). Candida infective endocarditis. Eur. J. Clin. Microbiol. Infect. Dis..

[28] Rivoisy C., Vena A., Schaeffer L., Charlier C., Fontanet A., Delahaye F., Bouza E., Lortholary O., Munoz P., Lefort A. (2018). Prosthetic Valve Candida spp. Endocarditis: New Insights into Long-term Prognosis—The ESCAPE Study. Clin. Infect. Dis..

[29] Giuliano S., Guastalegname M., Russo A., Falcone M., Ravasio V., Rizzi M., Bassetti M., Viale P., Pasticci M.B., Durante-Mangoni E. (2017). Candida endocarditis: Systematic literature review from 1997 to 2014 and analysis of 29 cases from the Italian Study of Endocarditis. Expert Rev. Anti Infect. Ther..

[30] Pierrotti L.C., Baddour L.M. (2002). Fungal endocarditis, 1995–2000. Chest.

[31] Ellis M.E., Al-Abdely H., Sandridge A., Greer W., Ventura W. (2001). Fungal endocarditis: Evidence in the world literature, 1965–1995. Clin. Infect. Dis..

[32] Delgado V., Marsan N.A., de Waha S., Bonaros N., Brida M., Burri H., Caselli S., Doenst T., Ederhy S., Erba P.A. (2023). 2023 ESC Guidelines for the management of endocarditis. Eur. Heart J..

[33] Steinbach W.J., Perfect J.R., Cabell C.H., Fowler V.G., Corey G.R., Li J.S., Zaas A.K., Benjamin D.K. (2005). A meta-analysis of medical versus surgical therapy for Candida endocarditis. J. Infect..

[34] Meena D.S., Kumar D., Agarwal M., Bohra G.K., Choudhary R., Samantaray S., Sharma S., Midha N., Garg M.K. (2022). Clinical features, diagnosis and treatment outcome of fungal endocarditis: A systematic review of reported cases. Mycoses.

[35] McCormack J., Pollard J. (2011). Aspergillus endocarditis 2003–2009. Med. Mycol..

[36] Pappas P.G., Kauffman C.A., Andes D.R., Clancy C.J., Marr K.A., Ostrosky-Zeichner L., Reboli A.C., Schuster M.G., Vazquez J.A., Walsh T.J. (2015). Clinical Practice Guideline for the Management of Candidiasis: 2016 Update by the Infectious Diseases Society of America. Clin. Infect. Dis..

[37] Kalokhe A.S., Rouphael N., El Chami M.F., Workowski K.A., Ganesh G., Jacob J.T. (2010). Aspergillus endocarditis: A review of the literature. Int. J. Infect. Dis..

[38] Reischies F., Hoenigl M. (2014). The role of surgical debridement in different clinical manifestations of invasive aspergillosis. Mycoses.

[39] Ryu K.M., Seo P.W., Kim S.-H., Park S., Ryu J.-W. (2009). Surgical treatment of native valve aspergillus endocarditis and fungemic vascular complications. J. Korean Med. Sci..

[40] De Pauw B., Walsh T.J., Donnelly J.P., Stevens D.A., Edwards J.E., Calandra T., Pappas P.G., Maertens J., Lortholary O., Kauffman C.A. (2008). Revised definitions of invasive fungal disease from the European Organization for Research and Treatment of Cancer/Invasive Fungal Infections Cooperative Group and the National Institute of Allergy and Infectious Diseases Mycoses Study Group (EORTC/MSG) Consensus Group. Clin. Infect. Dis..

[41] Pettersson G.B., Hussain S.T. (2019). Current AATS guidelines on surgical treatment of infective endocarditis. Ann. Cardiothorac. Surg..

[42] Baddour L.M., Wilson W.R., Bayer A.S., Fowler V.G., Tleyjeh I.M., Barsic B., Lockhart P.B., Gewitz M.H., Levison M.E., Bolger A.F. (2015). Infective endocarditis in adults: Diagnosis, antimicrobial therapy, and management of complications: A scientific statement for healthcare professionals from the American Heart Association. Circulation.

[43] Chen S.C.-A., Perfect J., Colombo A.L., Cornely O.A., Groll A.H., Seidel D., Albus K., de Almedia J.N., Garcia-Effron G., Gilroy N. (2021). Global guideline for the diagnosis and management of rare yeast infections: An initiative of the ECMM in cooperation with ISHAM and ASM. Lancet Infect. Dis..

[44] Jamil Y., Akinleye A., Mirzaei M., Lempel M., Farhat K., Pan S. (2023). Candida endocarditis: Update on management considerations. World J. Cardiol..

[45] Patterson T.F., Thompson G.R., Denning D.W., Fishman J.A., Hadley S., Herbrecht R., Kontoyiannis D.P., Marr K.A., Morrison V.A., Nguyen M.H. (2016). Practice guidelines for the diagnosis and management of aspergillosis: 2016 update by the infectious diseases society of America. Clin. Infect. Dis..

[46] Cheng J., Han H., Kang W., Cai Z., Zhan P., Lv T. (2024). Comparison of antifungal drugs in the treatment of invasive pulmonary aspergillosis: A systematic review and network meta-analysis. Front. Microbiol..

[47] Jenks J.D., Salzer H.J.F., Prattes J., Krause R., Buchheidt D., Hoenigl M. (2018). Spotlight on isavuconazole in the treatment of invasive aspergillosis and mucormycosis: Design, development, and place in therapy. Drug Des. Dev. Ther..

[48] Mulè A., Rossini F., Sollima A., Lenzi A., Fumarola B., Amadasi S., Chiari E., Lorenzotti S., Saccani B., Van Hauwermeiren E. (2023). Trichosporon asahii Infective Endocarditis of Prosthetic Valve: A Case Report and Literature Review. Antibiotics.

[49] Peinado-Acevedo J.S., Ramírez-Sánchez I.C. (2021). Endocarditis by Fusarium keratoplasticum. Mycopathologia.

[50] Bourlond B., Cipriano A., Regamey J., Papadimitriou-Olivgeris M., Kamani C., Seidel D., Lamoth F., Muller O., Yerly P. (2022). Case report: Disseminated Scedosporium apiospermum infection with invasive right atrial mass in a heart transplant patient. Front. Cardiovasc. Med..

[51] Chen C., Huang C.-H., Chen Y.-C. (2017). Timing of Surgery for Fungal Infective Endocarditis. Heart Surg. Forum.

[52] Edpuganti S. (2025). Fungal endocarditis: Microbial insights, diagnostic and therapeutic challenges in the modern era. Open Explor..

[53] Kurland S., Furebring M., Löwdin E., Olaison L., Sjölin J. (2025). Antifungal Therapy in Candida Infective Endocarditis: A Comparison of Echinocandins and Other Treatment Regimens in a Nationwide Cohort Study. Clin. Infect. Dis..

[54] Ben-Ami R., Bassetti M., Bouza E., Kosman A., Vena A. (2025). Candida endocarditis: Current perspectives on diagnosis and therapy. Clin. Microbiol. Infect..

[55] Thompson G.R., Soriano A., Skoutelis A., Vazquez J.A., Honore P.M., Horcajada J.P., Spapen H., Bassetti M., Ostrosky-Zeichner L., Das A.F. (2021). Rezafungin Versus Caspofungin in a Phase 2, Randomized, Double-blind Study for the Treatment of Candidemia and Invasive Candidiasis: The STRIVE Trial. Clin. Infect. Dis..

[56] Thompson G.R., Soriano A., Cornely O.A., Kullberg B.J., Kollef M., Vazquez J., Honore P.M., Bassetti M., Pullman J., Chayakulkeeree M. (2023). Rezafungin versus caspofungin for treatment of candidaemia and invasive candidiasis (ReSTORE): A multicentre, double-blind, double-dummy, randomised phase 3 trial. Lancet.

[57] Smith H.L., Bensman T.J., Mishra S., Li X., Dixon C.A., Sheikh J., McMaster O.G., Joshi A., Rubin D.B., Goodwin A. (2024). Regulatory Considerations in the Approval of Rezafungin (Rezzayo) for the Treatment of Candidemia and Invasive Candidiasis in Adults. J. Infect. Dis..

[58] Cornely O.A., Dupont H., Mikulska M., Rautemaa-Richardson R., Garcia-Vidal C., Thompson G.R., Hoenigl M. (2025). Rezafungin in special populations with candidaemia and/or invasive candidiasis. J. Infect..

[59] Trapani F., Viceconte G., Morena V., Tiseo G., Mori G., Kölking B., Khatamzas E. (2025). Long-term Safety and Effectiveness of Rezafungin Treatment in Candidemia and Invasive Candidiasis: Results From an Early Access Program in Italy and Germany. Open Forum Infect. Dis..

[60] Ponta G., Morena V., Strano M., Molteni C., Pontiggia S., Cavalli E.M., Grancini A., Mauri C., Castagna A., Galanti A. (2024). Safety of rezafungin as a long-term treatment option in two patients with complicated fungal infections: Two cases from Lecco Hospital (Italy). Antimicrob. Agents Chemother..

[61] Chandramohan D., Aguilar S., Gawrys G., Wiederhold N.P., Traugott K., Patterson T.F. (2025). A case of recurrent Candida glabrata fungemia and successful treatment with rezafungin. IDCases.

[62] Mori G., Gottardi M., Guffanti M., Castagna A., Lanzafame M. (2024). Treatment of Candida glabrata native valve endocarditis with rezafungin: A case report. JAC-Antimicrobial Resist..

[63] Forrister N.M., McCarty T.P., Pappas P.G., Shields R.K. (2024). New Perspectives on Antimicrobial Agents: Rezafungin. Antimicrob. Agents Chemother..

[64] El Ayoubi L.W., Allaw F., Moussa E., Kanj S.S. (2024). Ibrexafungerp: A narrative overview. Curr. Res. Microb. Sci..

[65] Pappas P.G., Cornely O., Koehler P., McCarty T.P., Alexander B.D., Miller R., Vazquez J.A., Sanders J.W., Morse C., Ostrosky-Zeichner L. (2021). 123. Oral Ibrexafungerp Outcomes by Fungal Disease in Patients from an Interim Analysis of a Phase 3 Open-label Study (FURI). Open Forum Infect. Dis..

[66] Open-Label Study to Evaluate the Efficacy and Safety of Oral Ibrexafungerp (SCY-078) in Patients with Candidiasis Caused by Candida Auris (CARES). https://ctv.veeva.com/study/open-label-study-to-evaluate-the-efficacy-and-safety-of-oral-ibrexafungerp-scy-078-in-patients-wit.

[67] Vanbiervliet Y., Van Nieuwenhuyse T., Aerts R., Lagrou K., Spriet I., Maertens J. (2024). Review of the novel antifungal drug olorofim (F901318). BMC Infect. Dis..

[68] Maertens J.A., Thompson G.R., Spec A., Donovan F.M., Hammond S.P., Bruns A.H.W., Rahav G., Shoham S., Johnson R., Rijnders B. (2025). Olorofim for the treatment of invasive fungal diseases in patients with few or no therapeutic options: A single-arm, open-label, phase 2b study. Lancet Infect. Dis..

[69] Shaw K.J., Ibrahim A.S. (2020). Fosmanogepix: A Review of the First-in-Class Broad Spectrum Agent for the Treatment of Invasive Fungal Infections. J. Fungi.

[70] Dhakal B.P., Tribble C.G., Bergin J.D., Winfrey S., Carter W.H. (2015). Recurrent candida prosthetic endocarditis over fifteen years managed with medical therapy and four valvular surgeries: A case report and review of literature. J. Cardiothorac. Surg..

[71] Melgar G.R., Nasser R.M., Gordon S.M., Lytle B.W., Keys T.F., Longworth D.L. (1997). Fungal prosthetic valve endocarditis in 16 patients an 11-year experience in a tertiary care hospital. Medicine.

[72] Ioannou P., Volosyraki M., Mavrikaki V., Papakitsou I., Mathioudaki A., Samonis G., Kofteridis D.P. (2020). Candida parapsilosis endocarditis. Report of cases and review of the literature. GERMS.

[73] Smego R.A., Ahmad H. (2011). The role of fluconazole in the treatment of candida endocarditis: A meta-analysis. Medicine.

